# Data analysis of the U–Pb geochronology and Lu–Hf system in zircon and whole-rock Sr, Sm–Nd and Pb isotopic systems for the granitoids of Thailand

**DOI:** 10.1016/j.dib.2018.10.176

**Published:** 2018-11-09

**Authors:** Romana E.C. Dew, Simon Nachtergaele, Alan S. Collins, Stijn Glorie, John Foden, Johan De Grave, Morgan L. Blades, Christopher K. Morley, Noreen J. Evans, Brandon L. Alessio, Pitsanupong Kanjanapayont, Rosalind King, Punya Charusiri

**Affiliations:** a Centre for Tectonics, Resources and Exploration (TRaX), Department of Earth Sciences, The University of Adelaide, Adelaide, South Australia 5005, Australia; b PTTEP, EnCo, Soi 11, GGS, Vibhavadi Rangsit Road, Chatuchak, Bangkok 10900, Thailand; c Department of Geological Sciences, Chiang Mai University, 239 Huay Kaew Road, Chiang Mai 50200, Thailand; d Department of Geology, Ghent University, Krijgslaan 281.S8, WE13, 9000 Ghent, Belgium; e School of Earth and Planetary Science, John de Laeter Centre, TIGeR, Curtin University, Perth, Western Australia, 6102, Australia; f Basin Analysis and Structural Evolution Special Task Force for Activating Research (BASE STAR), Department of Geology, Faculty of Science, Chulalongkorn University, Bangkok 10330, Thailand

## Abstract

This data article provides zircon U–Pb and Lu–Hf isotopic information along with whole-rock Sm–Nd, Sr and Pb isotopic geochemistry from granitoids in Thailand. The U–Pb ages are described and the classification of crystallisation and inherited ages are explained. The petrography of the granitoid samples is detailed. The data presented in this article are interpreted and discussed in the research article entitled “Probing into Thailand’s basement: New insights from U–Pb geochronology, Sr, Sm–Nd, Pb and Lu–Hf isotopic systems from granitoids” (Dew et al., 2018).

**Specifications table**

**Table:** 

Subject area	*Geology*
More specific subject area	*Geochronology, isotope geochemistry, zircon, granitoid*
Type of data	*Tables, images (photographs and microscopy)*
How data was acquired	*Petrography and Thin Section Imagery: Zeiss AxioScope A1 microscope with a AxioCam MRc5 camera*
	*Cathodoluminescence:* FEI Quanta600 Scanning Electron Microscope with the Mineral Liberation Analysis
	*U–Pb geochronology: Agilent 7900x with a New Wave NW213 laser ablation system with a TwoVol2 sample chamber (Adelaide Microscopy)*
	*Lu–Hf isotope analyses: Resonetics S-155-LR 193nm excimer laser ablation system connected to a Nu Plasma II multi-collector ICP–MS (Perth) and a Neptune Plus multi-collector ICP–MS (Wollongong)*
	*Whole-rock geochemistry: Finnigan MAT262 thermal ionization mass spectrometer (University of Adelaide Isotope Geochemistry Facility)*
Data format	*The 2015 U–Pb data were collected, corrected and filtered in the GLITTER version 3.0 software package**[2]**. The 2017* U–Pb *data and all* Lu–Hf analyses *were reduced using Iolite software**[3]*.
	*Corrected individual analyses are given in .xlsx format*
Experimental factors	*Zircons were extracted from bulk rock granitoid samples through the conventional magnetic and heavy liquid separation procedures.*
	*Whole-rock geochemistry samples were prepared with standard milling equipment.*
Experimental features	*Separated zircon grains were mounted in epoxy resin, polished and mapped by cathodoluminescence.*
	*Major and trace elemental compositions were determined prior to whole-rock isotopic analyses using an x-ray fluorescence spectrometer.*
Data source location	*Granitoid belts of Thailand, specific coordinates for each sample tabulated.*
Data accessibility	*Data is with this article and/or the below related research article*
Related research article	[1] Dew, R.E.C., Collins, A.S., Glorie, S., Morley, C.K., Nachtergaele, S., Blades, M.L., King, R., Foden, J., De Grave, J., Kanjanapayont, P., Evans, N.J., Alessio, B.L. and Charusiri, P. Probing into Thailand’s basement: New insights from U–Pb geochronology, Sr, Sm–Nd, Pb and Lu–Hf isotopic systems from granitoids. *Lithos*. 320-321, 332-354, https://doi.org/10.1016/j.lithos.2018.09.019.

**Value of the data**

• Assists in the understanding of the basement of Thailand, which is mostly covered by thick sequences of younger sediment.
• This combination of isotopic data is an innovative approach to investigate the buried rock systems that make up this key part of Southeast Asia.
• This approach and methodology can be implemented for future studies to further understand the evolution of this part of the world.
• Can be compared with other isotopic data from Southeast Asia for further insight into the tectonic history of the region.
• Provides information about Thailand’s history and its links to Australia

## 1 Data

In this data article, we report isotopic data from Thai granitoids of the Southeast Asian Granitoid Belts. This data includes U*–*Pb geochronology and Lu*–*Hf analyses from over 480 zircons and Sm–Nd, Sr and Pb isotopic geochemistry from 14 whole-rock granitoid samples.

U*–*Pb data were obtained during nine sessions along with common zircon reference materials (e.g., GJ-1, Plešovice and 91500). Lu*–*Hf data were obtained during three sessions along with common zircon reference materials (e.g., Mudtank and Plešovice). The zircon dataset contains the LA–ICP–MS raw and processed data. Each of the four whole-rock isotopes were measured for all samples in one TIMS analytic run. The mass fractionation for Sm–Nd, Sr, and Pb was controlled by the G-2 standard [4], with the BHVO-2 standard [5] was also used for the Sm and Nd analyses.

## 2 Experimental design, materials, and methods

### 2.1 Sample locations, affinity, and petrography

**Fig. 1:**
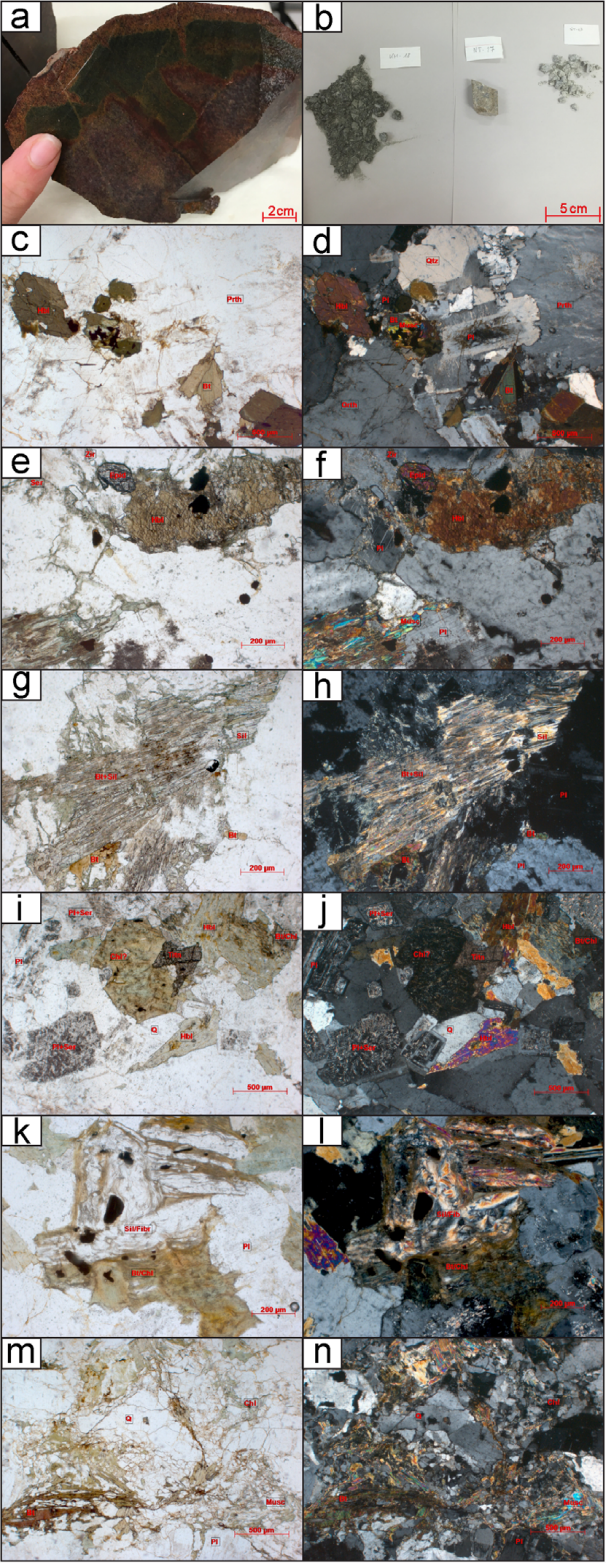

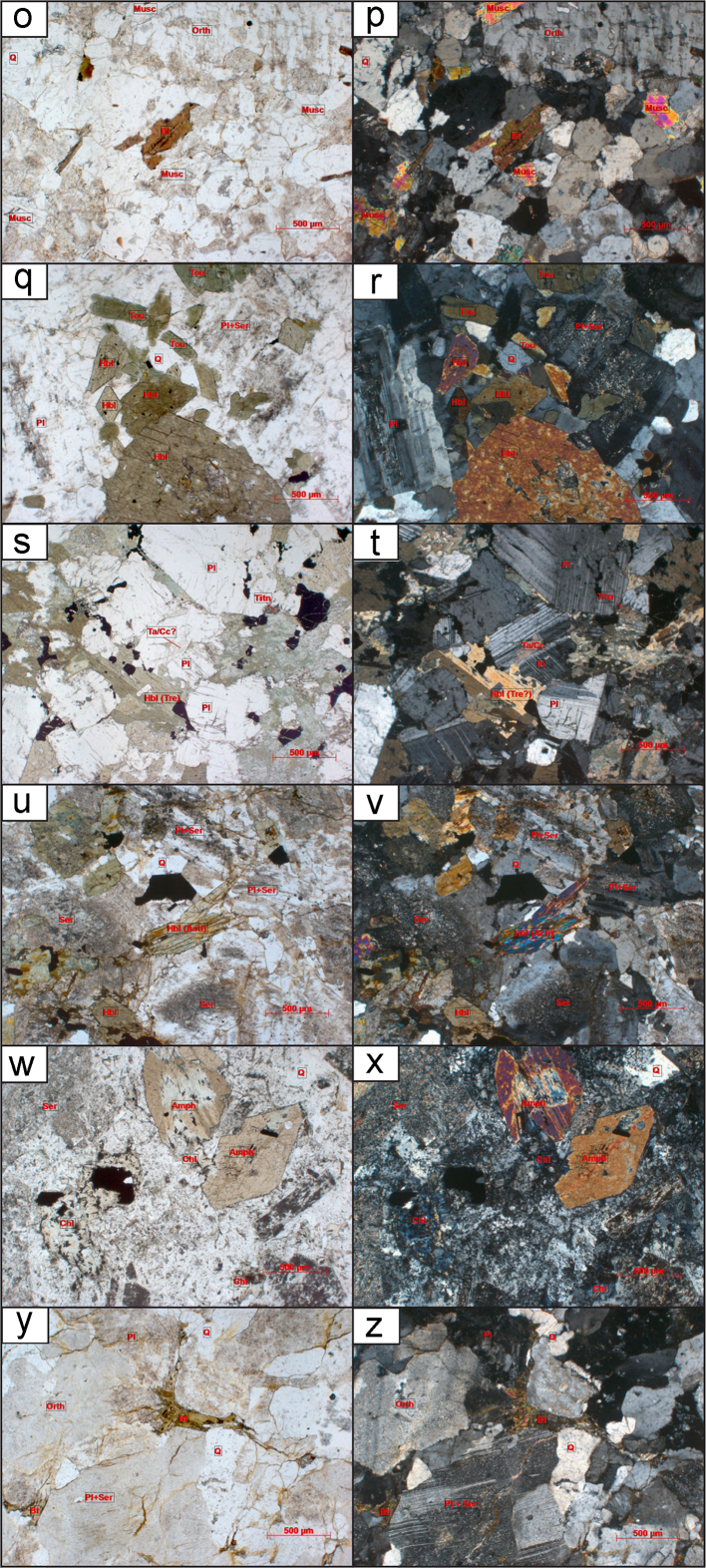

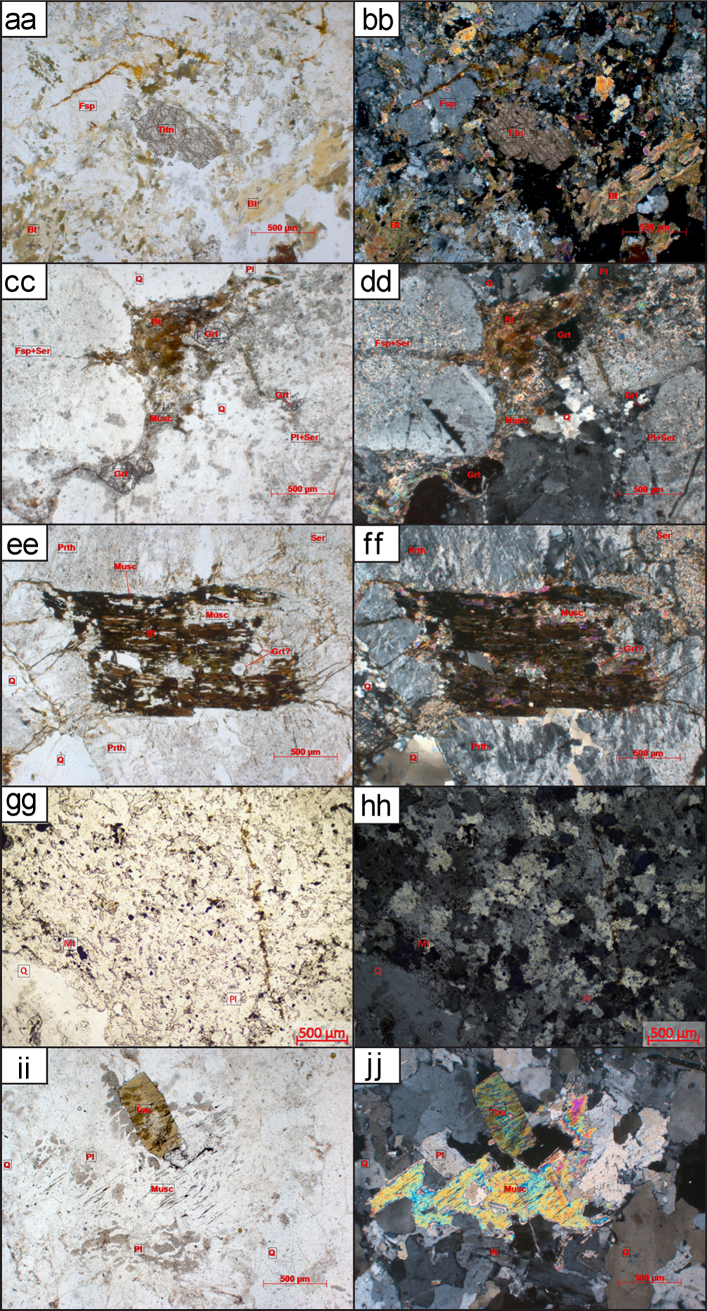

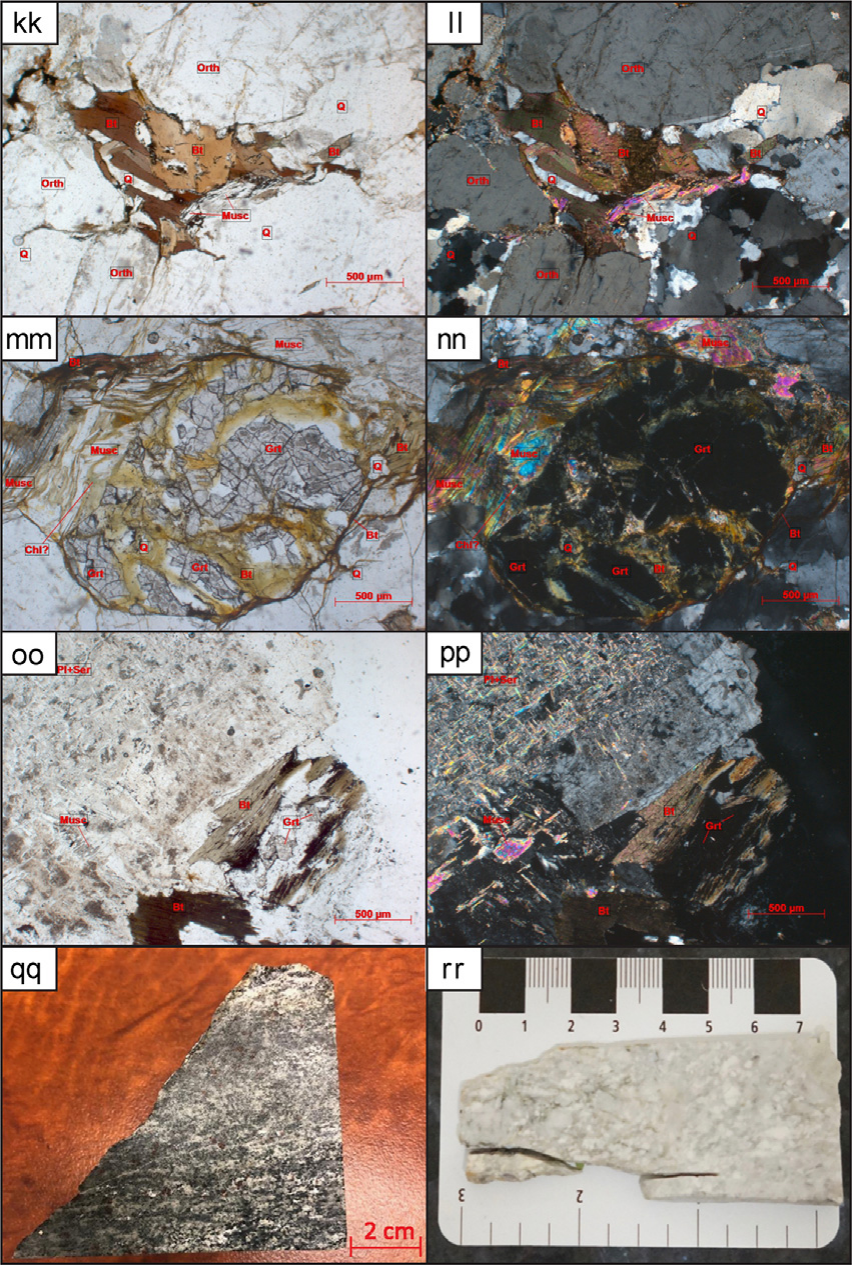
Thin sections, outcrop and hand specimens of granitoid samples. Minerals present are labelled where: Qtz is quartz; Bt is biotite; Mu is muscovite; Am is amphibole mainly hornblende in these samples; Pl is plagioclase; Mc is microcline and Px is pyroxene. (a) Hand specimen of RDT15_076A altered granodiorite (b) From left to right: rock chips of KM-18, NT-17 and ST-03. (c) KM-12 overview thin section image in Plane Polarised Light (PPL) 10x (d) KM-12 overview thin section image in Cross Polarised Light (XPL), (e) KM-25 overview thin section image in PPL, (f) KM-25 overview thin section image in XPL, (g) KM-25 overview thin section image showing sillimanite mineral growth in PPL, (h) KM-25 overview thin section image showing sillimanite mineral growth in XPL, (i) KM-26 overview thin section image in PPL, (j) KM-26 overview thin section image in XPL, (k) KM-26 overview thin section image showing sillimanite mineral growth in PPL, (l) KM-26 overview thin section image showing sillimanite mineral growth in XPL, (m) KM-40A overview thin section image in PPL, (n) KM-40A overview thin section image in XPL, (o) NT-01 overview thin section image in PPL, (p) NT-01 overview thin section image in XPL, (q) NT-04 overview thin section image in PPL, (r) NT-04 overview thin section image in XPL, (s) NT-06 overview thin section image in PPL, (t) NT-06 overview thin section image in XPL, (u) NT-07 overview thin section image in PPL, (v) NT-07 overview thin section image in XPL, (w) NT-09 overview thin section image in PPL, (x) NT-09 overview thin section image in XPL, (y) NT-10 overview thin section image in PPL, (z) NT-10 overview thin section image in XPL, (aa) NT-11 overview thin section image in PPL, (bb) NT-11 overview thin section image in XPL, (cc) NT-12 overview thin section image in PPL, (dd) NT-12 overview thin section image in XPL, (ee) NT-13 overview thin section image in PPL, (ff) NT-13 overview thin section image in XPL, (gg) RDGEOCHEM-1 overview thin section image in PPL, (hh) RDGEOCHEM-1 overview thin section image in XPL, (ii) ST-03 overview thin section image in PPL, (jj) ST-03 overview thin section image in XPL, (kk) ST-08A overview thin section image in PPL, (ll) ST-08A overview thin section image in XPL, (mm) ST-16 overview thin section image in PPL, (nn) ST-16 overview thin section image in XPL, (oo) ST-49A overview thin section image in PPL, (pp) ST-49A overview thin section image in XPL, (qq) RDT16_044 hand specimen, (rr) RDT16_053 hand specimen.

Twenty-nine granitoid samples in total are used for this study. For detailed preparatory methodology of the KM, ST, and NT samples see [6], [7]. The individual sample locations, lithology, associated granitoid belt where applicable and analysis method for each sample are outlined in Table 1 of [1]. Details of the petrography including mineralogy, textures and degree of deformation are outlined in Table 1. Hand specimen and thin section imagery of the analysed samples are displayed in Fig. 1. Some of the analysed granitoids are associated with named batholiths or specific plutons whose petrography and petrogenesis has been previously described, for further information see Table 1 of [1]. The sampling strategy for this study was to collect granitoids from all three terranes and across major faults and sutures to better delineate tectonic boundaries. Representative samples of granitoids, and therefore also their underlying basement, were collected from widespread localities within Thailand.

The three main tectonic domains in Thailand: Sibumasu, Sukhothai and Indochina, are associated with three large granites provinces: Western Thailand/Myanmar, North Thailand–West Malaya Main Range and East Malaya, for further information and locations see [8], [9], [10]. The Western Thailand–Myanmar/Burma province also known as the Mogok–Mandalay–Mergui Belt extends from eastern Myanmar (Burma) southwards to Phuket Island. The Mogok–Mandalay–Mergui Belt and the Western Thailand North Thailand–West Malaya Main Range broadly correlate with the Sibumasu Terrane and Inthanon Zone respectively although this correlation depends on the delineation of the terrane boundaries, which are have been variably defined in the past [9], [11], [12], [13]. The mineralogy consists of hornblende–biotite I-type granodiorite–granites and felsic biotite–K-feldspar (± garnet ± tourmaline) S-type granites [10], [14]. These granitoids are associated with abundant tin mineralisation in greisen type veins [10]. Recent U–Pb dating of Western Thailand granitoids in Phuket displays zircons with Triassic cores (e.g., 212 ± 2 Ma and 214 ± 2 Ma) and Cretaceous rims (81.2 ± 1.2 and 85–75 Ma) [10].

The North Thailand–West Malaya or Main Range province occurs northward from West Malaya towards to the Doi Inthanon range of north Thailand. This province is characteristically is composed of biotite–K-feldspar S-type granites although also contains subordinate I-type granitoids [8], [10]. Searle et al. [10] suggest that they are more likely to be evolved felsic I-types rather than the S-type granites proposed by Cobbing et al. [8]. The ages of this province range from early late Triassic to late early Jurassic [14]. The sheer batholithic proportions of this province suggest crustal anatexis as the potential source [10]. It is suggested that these granitoids are orogenic, forming as a result of the crustal thickening following the closure of the Paleo-Tethys and the collision of Sibumasu and Sukhothai-Indochina in the late Triassic [10], [14]. The boundary between the Western province and the West Malaya Main Range has been historically defined as the Paleogene Khlong Marui fault (e.g., [8]). However, Searle et al. [10] suggest that the nature of the boundary is not as clear as stated by Cobbing et al. [8] due to the presence of S-type granite either side of the Khlong Marui fault, which also formed later than the granite emplacement.

The Eastern or East Malaya granitoid province is mostly Permo-Triassic I-type granites, granodiorites and tonalities but with subordinate S-type plutons and A-type syenite–gabbros [8], [10]. The I-type granitoids of the East Malaya province are distributed throughout the Sukhothai and Indochina terranes [9], [14]. These granitoids are thought to originate from arc magmatism caused by the subduction of the Paleo-Tethys under Indochina [10], [14]. However, the genesis of the S-type granitoids associated with the Loei-Phetchabun Volcanic Belt is unlikely to be subduction-induced arc magmatism. Instead, it is suggested by Sone and Metcalfe [14] to be associated to the crustal thickening of western Indochina that was induced by back-arc compression and later emphasised by the Sibumasu collision. The East Malaya province granitoids range primarily from the early Permian to end of the Triassic with occasional Cretaceous magmatism, which is shared with the Western Thailand–Myanmar/Burma granitoid province [10], [14].

### 2.2 Zircon sample preparation

Eighteen granitoid rock samples were crushed and sieved for collecting zircon grains through the conventional magnetic and heavy liquid separation procedures. About 50 randomly selected zircon grains were set in epoxy resin and polished for U–Pb zircon age analysis. In order to characterise the textural and chemical-zoning features within each zircon (Table 2), cathodoluminescence (CL) images for zircon were obtained using the FEI Quanta600 Scanning Electron Microscope with the Mineral Liberation Analysis (MLA) at Adelaide Microscopy, Adelaide, South Australia. Each mount was carbon coated prior to CL imaging to increase the conductivity of the sample and to retrieve higher quality images.

For each sample, 20–30 magmatic grains were selected for laser ablation inductively coupled plasma mass spectrometry (LA–ICP–MS) at Adelaide Microscopy, Adelaide, South Australia. All assumed inherited grains or distinct zircon domains were also ablated using LA–ICP–MS. The analyses were conducted on an *Agilent 7900x* with a *New Wave NW213* laser ablation system with a TwoVol2 sample chamber. A 30 µm spot size was used where possible, however, one analysis session used a smaller 25 µm spot size due to the smaller size of the zircon grains (see Table 3). A 5 Hz pulse rate was used with a typical pit depth of 30–50 µm. For further details of the analytical methodology of this laboratory’s technique see [15]. The exact fluence settings across the two-year analysis period ranged from 5.6 to 7 J/cm^2^. The isotopes measured for all analyses were ^204^Pb, ^206^Pb, ^207^Pb, ^208^Pb, ^232^Th and ^238^U (see Table 3). The GEMOC zircon standard GJ-1 (^207^Pb/^206^Pb TIMS age of 607.7 ± 4.3 Ma, ^206^Pb/^238^U TIMS age of 600.7 ± 1.1 Ma and ^207^Pb/^235^U age of 602.0 ± 1.0 Ma all ages given as 2σ; [16]) was run as the primary standard every 10–20 unknown analyses, to correct for isotopic drift and down-hole fractionation. The Plešovice zircon standard (^206^Pb/^238^U age of 337.13 ± 0.37 Ma (2σ); [17]) was analysed as a secondary standard to check the accuracy of the technique. Across all analytical sessions, analyses of GJ-1 yielded a ^206^Pb/^238^U weighted average age of 601.61 ± 0.47 Ma (*n*=556, MSWD=1.10, 2σ) and Plešovice yielded a ^206^Pb/^238^U weighted average age of 339.55 ± 0.73 (*n*=243, MSWD=3.7, 2σ). For the later batches analysed in 2017, ^90^Zr and ^202^Hg isotopes were measured where possible, see Table 3. For some LA–ICP–MS runs, ^204^Pb was not measured due to the unresolvable isobaric interference from ^204^Hg [18]. For two of the analysis sessions, the rare earth element suite was also monitored (see Table 3). The 2017 batches were also run with the 91500 zircon as another secondary standard (^207^Pb/^206^Pb age of 1065 Ma and ^206^Pb/^238^U age of 1062 Ma; [19]). During these analyses, 135 analyses of 91500 yielded a ^206^Pb/^238^U age of 1036.2 ± 3.3 (MSWD=4.2, 2σ). Two different 91500 crystals were used for these analyses, where one crystal gives good reproducibility while the other is more heterogeneous. This heterogeneity accounts for the large MSWD across the three analytic runs monitoring this standard. However, in this study GJ-1 was used as the primary zircon standard so the isotopic drift across the analytic periods in the secondary standards (Plešovice and 91500 was used as one of two secondary standards and was not used as a primary standard to correct for isotopic drift and down-hole fractionation.

The 2015 data were collected, corrected and filtered in the GLITTER version 3.0 software package [2]. The 2017 data were reduced using Iolite software [3]. All concordia diagrams and weighted averages were calculated using ISOPLOT 4.15 for Excel [20].

### 2.3 Zircon Lu–Hf isotope analysis

After the U–Pb zircon geochronology analyses were conducted, 10 of these granitoid samples were selected for further zircon Lu–Hf isotope analysis. A subset of the zircon grains for each sample were then analysed for their hafnium isotopic composition (the specific grains analysed and their concordance percentage are highlighted in Table 4). The number of Hf analyses for each sample was determined by the variability in the age data and the amount of interpreted inheritance. Nine samples used for Hf and Lu isotopic analyses (all samples except KM-20) were analysed using a *Resonetics S-155-LR* 193 nm excimer laser ablation system connected to a *Nu Plasma II* multi-collector ICP–MS in the GeoHistory Facility, John de Laeter Centre, Curtin University, Perth, Western Australia. Analyses were carried out using a laser beam diameter of 50 μm. After two cleaning pulses and 40 s of baseline acquisition, zircon grains were ablated for 35 s using a 10 Hz repetition rate, and a laser beam energy of 2 J/cm^2^. All isotopes (^180^Hf, ^179^Hf, ^178^Hf, ^177^Hf, ^176^Hf, ^175^Lu, ^174^Hf, ^173^Yb, ^172^Yb and ^171^Yb) were counted on the Faraday collector array (see Table 4 and 5 for further details). Time resolved data were baseline subtracted and reduced using Iolite (DRS after [21]) where ^176^Yb and ^176^Lu were removed from the 176 mass signal using ^176^Yb/^173^Yb = 0.7962 (and ^176^Lu/^175^Lu = 0.02655 [22]) with an exponential law mass bias correction assuming ^172^Yb/^173^Yb = 1.35274 as per [22]. The interference corrected ^176^Hf/^177^Hf was normalised to ^179^Hf/^177^Hf = 0.7325 [23], for mass bias correction. Mud Tank (^176^Hf/^177^Hf of 0.282505 ± 0.000044 [24]) was used as the primary standard for Hf isotopes and R33 as the primary standard for Lu–Hf analyses (^176^Hf/^177^Hf of 0.282764 ± 0.000014 [25], Lu/Hf ratios of 0.001989 ± 0.000869 [17]).

Mudtank analyses yielded ^176^Hf/^177^Hf weighted-means of 0.2825064 ± 0.0000097 (2σ, *n*=29, MSWD=0.89, for the first session including all Hf samples except RDT16_053 and KM-20; Table 5) and 0.2825071 ± 0.0000081 (2σ, *n*=29, MSWD=0.041, for RDT16_053 analysis run). The R33 standard yielded ^176^Hf/^177^Hf weighted-mean of 0.282718 ± 0.000010 (2σ, *n*=28, MSWD=1.14) and Lu/Hf ratios of 0.0019903 ± 0.0000097 (2σ, *n*=28, MSWD=19) for the first session (including all Hf samples except RDT16_053 and KM-20) and for the analyses for RDT16_053 yielded ^176^Hf/^177^Hf weighted-mean of 0.282708 ± 0.000017 (*n*=14, MSWD = 3.6) and Lu/Hf ratios of 0.001988 ± 0.000040 (2σ, *n*=28, MSWD=101). Secondary standards were 91500 (^176^Hf/^177^Hf of 0.282306 ± 0.00004 [21]), GJ-1 (^176^Hf/^177^Hf of 0.282000 ± 0.000005 [26]) and FC-1 (^176^Hf/^177^Hf of 0.282172 ± 0.000042 [24]). Additionally for the first session (March 2017 Table 5, including all Hf samples except RDT16_053 and KM-20), Plešovice was used as a secondary standard (^176^Hf/^177^Hf of 0.282482 ± 0.000013 and Lu/Hf ratio of 0.0004–0.0015 [17]). The ^176^Hf/^177^Hf weighted averages for the analysis of the secondary standards are outlined in Table 5. The corrected ^178^Hf/^177^Hf ratio was calculated to monitor the accuracy of the mass bias correction and yielded average values of 1.467219 ± 0.000016 (*n*=214; March 2017) and 1.467117 ± 0.000011 (*n*=296; July 2017), which are both within the range of values reported by [27].

Hafnium analysis for one sample (KM-20) was undertaken using a *Neptune Plus* multi-collector ICP–MS at the University of Wollongong, New South Wales. The dwell time was 50 s with 5 Hz repetition rate and an intensity of 4.4 J/cm^2^. Standards were Mudtank (^176^Hf/^177^Hf of 0.282505 ± 0.000044 [24]) and Plešovice (^176^Hf/^177^Hf of 0.282482 ± 0.000013 and Lu/Hf ratio of 0.0004–0.0015 [17]), yielding ^176^Hf/^177^Hf weighted-averages of 0.282473 ± 0.000023 (2σ, *n*=2) and 0.282450 ± 0.000018 (2σ, *n*=2) respectively. The corrected ^178^Hf/^177^Hf ratio was calculated to monitor the accuracy of the mass bias correction and yielded an average value of 1.467227 ± 0.000012 (*n*=19), which is within the range of values reported by [27]. All Lu–Hf data was reduced using Iolite software [3]. Calculation of εHf values employed the decay constant of [28] and the Chondritic Uniform Reservoir (CHUR) values of [29], depleted mantle Lu/Hf values of [30] and Hf/Hf values of [31].

### 2.4 Sm–Nd, Sr and Pb whole-rock geochemistry

Sm–Nd and Sr isotopic whole-rock analyses were conducted for 14 granitoid samples and one duplicate run for Sm–Nd analyses (NT-13) at the University of Adelaide’s Isotope Geochemistry Facility (see Table 1 of [1]). Eight samples and one duplicate (NT-13) were used for whole-rock Pb isotope measurements (see Table 1 of [1]). These samples were chosen due to their spatial distribution across the main tectonic terranes in Thailand and also containing Nd, Sr and Pb elemental concentrations above the detection limits of the X-ray fluorescence spectrometer (XRF) at Franklin and Marshall College, U.S.A. (major and trace element data provided in Table 6). A detailed methodology for the Sr and Sm–Nd whole-rock isotope techniques conducted in the same laboratory are outlined by [32].

To minimise error magnification, the optimal spike amount was calculated for each sample (i.e., approximately 0.4 g of Sm–Nd spike H per 2 µg Nd and 0.08 g of Sr spike C per 1 µg Sr). Six of the 14 samples used for Nd and Sr whole-rock geochemistry contained Pb ppm concentrations below the 1 ppm detection limit of the XRF, therefore, the subsequent whole-rock Pb TIMS analyses were not completed on these samples. The Pb isotopes were corrected for mass fractionation using the Southampton–Brest lead ^207^Pb–^204^Pb double spike (SBL74) as outlined by [33]. This spike was formulated to minimise uncertainty propagation with sample ^206^Pb/^204^Pb isotope compositions in the range of 14–30 [33]. SBL74 is calibrated relative to a conventional reference value ^208^Pb/^206^Pb = 1.00016 for NIST SRM 982 and has a composition of ^204^Pb/^206^Pb = 9.2317, ^207^Pb/^206^Pb = 36.6450 and ^208^Pb/^206^Pb = 1.8586 [33].

Lead contamination was effectively negated by removing metals and silicates and minimising atmospheric and procedural contamination by conducting analyses in a clean lab setting of the University of Adelaide’s Isotope Geochemistry Facility. Lead was isolated from the sample matrix by twice passing each sample through an HBr solution using anion exchange chromatography. For TIMS analysis, each sample was loaded with silicic acid–phosphoric acid emitter onto two zone-refined Re filaments using the double spike procedure outlined by [33]. The two filament loads consist of: a “natural” run with sample only and a sample–spike mixture run. The optimum mixture of sample and spike was calculated as ^204^Pb_Sample_/^204^Pb_Spike_ (*q*) = 0.09, with a tolerance range of 0.03–0.65 within which negligible uncertainty magnification was observed.

Sm–Nd, Sr and Pb whole-rock isotopes were measured on the *Isotopix Phoenix* thermal ionization mass spectrometer (TIMS) at the University of Adelaide, South Australia (see Table 7). The total procedural blanks were <475 pg for Sr, <221 pg for Sm, <699 pg for Nd and <74.1 pg for Pb. If the procedural blank is <1/1000th sample then it can be considered negligible (e.g., a procedural blank of 2 ng Nd could be considered not significant for a sample of 2 µg Nd; see Table 7 for elemental concentrations measured by TIMS for individual samples). The mass fractionation for Sm–Nd, Sr and Pb was controlled by the G-2 standard [4], which yielded average ratios of ^143^Nd/^144^Nd = 0.512261 ± 0.000002 (2 SE) and ^87^Sr/^86^Sr = 0.709770 ± 0.000003 (2 SE). For the Pb isotopes the G-2 standard [4], yielded corrected ratios of 18.3870, 15.6361 and 38.903 for ^206^Pb/^204^Pb, ^207^Pb/^204^Pb and ^208^Pb/^204^Pb, respectively. A secondary standard, BHVO-2 [5], was also measured for Sm and Nd analyses yielding average ratios of ^143^Nd/^144^Nd = 0.513034 ± 0.000002 (2 SE). Although none of the samples analysed are basaltic in composition, the Nd ppm value of the BHVO-2 basalt standard was similar to the expected Nd ppm concentration of the unknown granitoid samples. The Nd isotopic reference JNdi-1 [0.512115 ± 0.000007; 34], yielded average ratios of ^143^Nd/^144^Nd = 0.512100 ± 0.000002 (2 SE) and 0.512103 ± 0.000002 (2 SE). The University of Adelaide Isotope Geochemistry Facility’s laboratory average for the JNdi-1 Nd isotopic reference is 0.512106 ± 0.000009 (2σ). During the period of Sr analysis, the two analyses of the Sr isotopic standard SRM987 yielded average ratios of ^87^Sr/^86^Sr = 0.710246 ± 0.000003 (2 SE) and 0.710243 ± 0.00002 (2 SE). SRM981, the Pb isotopic reference used in this study (^206^Pb/^204^Pb = 16.9412 ± 0.0003; ^207^Pb/^204^Pb = 15.4988 ± 0.0006; ^208^Pb/^204^Pb = 36.7233 ± 0.0013 [33]), yielded corrected ratios of 16.9436, 15.5013 and 36.729 for ^206^Pb/^204^Pb, ^207^Pb/^204^Pb and ^208^Pb/^204^Pb respectively. The whole-rock measurements from the TIMS analyses and calculated initial isotopic values are displayed in Table 7.

### 2.5 U–Pb crystallisation and inherited age determination

The crystallisation ages have been interpreted individually for each sample, depending on the nature of the data. This is because different zircons behave differently in the U–Pb isotopic system. The data interpreted to represent the crystallisation age are stated in [1] with further explanatory information detailed below for each sample (the data are also highlighted in bold in Table 4). Inherited grains are defined as zircons that are older than the crystallisation age of the sample. The associated Concordia curves, regression lines and weighted average age plots are illustrated in Fig. 3 from [1].

#### 2.5.1 Sibumasu terrane

While the interpreted crystallisation age for ST-16 was Cambrian, there were also concordant ^206^Pb/^238^U ages between 371.5 ± 5.81 Ma and 83.7 ± 1.37 Ma, which may have been due to later resetting post-crystallisation (see Table 4). These younger analyses from ST-16 had very low Th:U, suggesting that the large Th ion has diffused from the zircon during a subsequent thermal event [see 35]. The observation that young ^206^Pb/^238^U age zircons have low Th/U ratios suggests that both Pb and Th have been lost from the zircon. This is supported by petrographic observations of igneous garnet breaking down to muscovite, chlorite and biotite (Fig. 1—mm, nn). Older concordant (±10%) ages were measured from ^207^Pb/^206^Pb Age of 3189.3 ± 17.83 Ma to ^206^Pb/^238^U Age of 710.5 ± 11.63 Ma (Table 1). The older ages analysed were interpreted to be inherited from events prior to the crystallisation of the granite.

NT-17 contained concordant ±5% zircon analyses with ages ranging from 196.3 ± 2.8 Ma and 209.7 ± 3.0 Ma (2σ). The measured dataset included several older analyses with ages of 2755 Ma, 2685 Ma, 1248 Ma, 947 Ma and 836 Ma (see Table 4). The Concordia of NT-17 (Fig. 3 of [1]) shows that many of the zircons analysed sit off the Concordia line, indicating that the U–Th–Pb system was no longer working as a closed system. A weighted average was first taken from all analyses within ±5% concordance, yielding a ^206^Pb/^238^U age of 211.2 ± 4.0 Ma (*n*=17, MSWD=27). This calculated age has a very large Mean Square Weighted Deviation (MSWD) indicating that the data are overdispersed with the observed data scatter exceeding the predicted analytical uncertainties. In attempt to better constrain the crystallisation age, another weighted average age was taken from the cluster of ten concordant (±5%) data analyses, which consequently yielded an age within error of the first weighted average and the data was more closely dispersed within the range of the predicted analytical uncertainties (see Table 4 and Fig. 3 of [1]).

An Upper Triassic crystallisation age was interpreted for ST-08A (see Table 4). Although the weighted average age MSWD of 3.1 indicates that the data are overdispersed, there are no clear distinguishing factors to filter the data any further. Two interpreted concordant inherited ages were found with a ^207^Pb/^206^Pb age of 2473 ± 34 Ma and a ^206^Pb/^238^U age of 393.1 ± 9.3 Ma.

Similar to ST-08A, the crystallisation age of ST-13 was also calculated to be Upper Triassic. Cretaceous aged analyses from ST-13 often had very low Th:U (Table 4, shown in white on Fig. 3 of [1]), suggesting that the large Th ion has diffused from the zircon during a subsequent thermal event. The observation that young ^206^Pb/^238^U age zircons have low Th/U ratios suggests that both Pb and Th have been lost from the zircon. Therefore, this younger cluster of Cretaceous data was interpreted to be the age of metamorphic resetting (see Table 4 and text and Fig. 3 of [1]). There was a prominence of age inheritance for ST-13 ranging from 1736 Ma to 500 Ma. Many of the older ages were taken from cores of zircons with multiple domains. ST-13 often up to four CL domains in the one zircon. Often at least one domain was Triassic in age, these inner domains were interpreted to be age of crystallisation.

The majority of zircon analyses for ST-49A were around 80 Ma, with 11 concordant (±5%) zircon analyses, yielding a ^206^Pb/^238^U age of 79.8 ± 1.6 Ma (*n*=11, MSWD=3.3; Table 4). This calculated age has a very large Mean Square Weighted Deviation (MSWD) indicating that the data are overdispersed with the observed data scatter exceeding the predicted analytical uncertainties. In attempt to better constrain the crystallisation age, another weighted average age was taken from the cluster of six concordant (±5%) data analyses yielding a much smaller MSWD of 0.37 (^206^Pb/^238^U age of 81.4 ± 1.1 Ma). The lower intercept of a common Pb regression trend of 78.9 ± 1.1 Ma (*n*=26, MSWD=2.2, Fig. 3 of [1]). All three of these calculated ages are within error of each other, however, the interpreted crystallisation age was determined to be the cluster of concordant (±5%) data analyses yielding a ^206^Pb/^238^U age of 81.4 ± 1.1 Ma (*n*=6, MSWD=0.37, data highlighted in Table 4). One zircon analysis within 10% concordance gave a ^206^Pb/^238^U age of 259.8 ± 4.1 Ma, which is interpreted as an inherited grain, possibly reflecting an earlier magmatic event in the region.

Like the crystallisation age of ST-49A, the interpreted crystallisation age for ST-18 was also determined to be Upper Cretaceous (see Table 4 for age data). Zircons from this sample have interpreted age inheritance with concordant ages spanning from 2729 Ma to 524 Ma.

No crystallisation age could be constrained from the zircon analyses conducted on RDT15_076A. It was interpreted that the calculated ages were all inherited zircons (Table 4). Interpreted concordant inherited ages range from ^207^Pb/^206^Pb age of 2462 ± 21 Ma to ^206^Pb/^238^U age of 448.1 ± 6.7 Ma (Table 4).

#### 2.5.2 Inthanon zone

Twenty-two concordant (±5) magmatic zircon analyses from Th11/02 were used to calculate the weighted average, which yielded an Upper Triassic crystallisation age (Table 4). A discordia line also gives a lower intercept within error of this weighted average at 206.7 ± 1.3 Ma (see Fig. 3 of [1]). The upper intercept of this regression line gives an age on the boundary of the Eoarchean and the Paleoarchean at 3617 ± 200 Ma. One discordant grain was found at 258.8 Ma, with two older grains within 10% discordance with ^206^Pb/^238^U ages of 409.4 ± 6.53 Ma and 304.8 ± 4.99 Ma. There was also a concordant (±5) inherited grain with a ^206^Pb/^238^U age of 562.9 ± 8.82 Ma.

The interpreted crystallisation age for RDT16_053 is the weighted average of all concordant (±5) analyses with Th:U>0.1, which also yielded an Upper Triassic age like Th11/02 (see Table 4 for reduced data). Analyses younger than 200 Ma were interpreted to have lost Th and Pb from a subsequent thermal event, which was supported by the Th:U and (^176^Hf/^177^Hf)_**i**_ values. Three older inherited cores are present with Paleoproterozoic–Neoarchean ^207^Pb/^206^Pb ages ranging from 2746 ± 29 Ma to 2430 ± 27 Ma. The inherited zircon ages found in this sample are similar those found in ST-18 from the Sibumasu Terrane.

The interpreted crystallisation age for RDT16_044 was is Upper Cretaceous in age (Table 4). This age was determined from a singular zircon analysis. The Th:U of this zircon is >0.1 however, the majority of the analyses from this sample have low Th:U indicating that both Pb and Th have been lost from the zircon during subsequent thermal events. Discordia could not be completed with Isoplot software since the youngest cluster of data containing positive and negative rho values.

#### 2.5.3 Sukhothai terrane

All five samples taken from the Sukhothai Terrane yielded magmatic ages within 13 Ma between 238.6 and 226.5 Ma (data are displayed in Table 4). No concordant inherited zircons were found from any of these samples (±5% discordant data are coloured yellow in Fig. 3 of [1]).

The oldest interpreted crystallisation age from the Sukhothai Terrane was interpreted from NT-12 (weighted average age of 238.0 ± 2.9 Ma, see Table 4). The lower concordia intercept of a common Pb regression line for NT-12 is also within error of this weighted average age and yields a ^206^Pb/^238^U age of 238.6 ± 3.1 Ma (*n*=12, MSWD=1.03).

The interpreted crystallisation age for NT-10 was Upper Triassic (a weighted average ^206^Pb/^238^U age of 236.6 ± 2.9 Ma, Table 4). Analyses from NT-10 also form a linear trend with a lower Concordia intercept at 236.2 ± 2.7 Ma and an upper intercept of 5107 ± 750 Ma (*n*=18, MSWD = 0.84) representing a common Pb component. The lower intercept of this trend is the same age as the weighted average age only the weighted average incorporates 0.2 more uncertainty (at 2σ).

Nineteen concordant (±5%) analyses from NT-11 with Th:U>0.1 yield a ^206^Pb/^238^U weighted average of 228.7 ± 2.1 Ma (MSWD=0.48; Table 4). No concordant inherited zircons were found, although two very discordant analyses had ^206^Pb/^238^U ages of 338.8 ± 5.66 Ma and 121.4 ± 2.08 Ma (see Table 4).

The magmatic age of Th11/01 is interpreted to be the weighted average of the ±5% concordant analyses with Th:U>0.1 yielding a ^206^Pb/^238^U age of 227.9 ± 1.9 Ma (*n*=23, MSWD=1.2; Table 4). Th11/01 contained two older very discordant analyses with ^206^Pb/^238^U ages of 455.6 ± 7.64 Ma and 316.1 ± 5.47 Ma (Table 4).

Concordant (±5%) analyses with Th:U>0.1 from sample NT-09 yielded a weighted average ^206^Pb/^238^U age of 226.5 ± 1.8 Ma (*n*=18, MSWD=0.48; Table 4). No concordant inherited zircons were found, although one very discordant analysis had a ^206^Pb/^238^U age of 692.4 ± 10.58 Ma.

#### 2.5.4 Indochina

KM-20 contains ^206^Pb/^238^U ages within ±5% discordance for the entire first half of the Triassic period, from 249.4 ± 5.9 Ma to 221.5 ± 5.7 Ma (see Table 4). We interpret that the Lower and Middle Triassic ages are inherited and that crystallisation age of this sample was calculated from the weighted average of all analyses from the youngest to oldest concordant zircon in the Upper Triassic (see Table 4).

The NT-07 quartz diorite sample, was collected further south 60 km east of Nakhon Sawan. The crystallisation age of this sample is interpreted to be Permian (see Table 4). It was very difficult to determine domains from the CL imaging since the majority of zircons from this sample contained dark homogeneous zones, sometimes the whole zircon was dark and homogeneous. Therefore, it was difficult to determine the domain being ablated. This sample yielded a spread of analyses that may suggest limited post-crystallisation disturbance of the isotopic system that is supported by the sericitisation of feldspars seen in thin section (Fig. 1—u, v).

### 2.6 Published isotopic data used for comparison

**Table:** 

Type and/or location of isotopic data	References for published data
Hafnium (Fig. 4 in [1])	[9], [36], [37], [38], [39], [40], [41], [42], [43], [44], [45]
εNd_(t)_ against age (Fig. 5a in [1])	[44], [46], [47], [48], [49].
εNd_(t)_ against initial Sr (Fig. 5c in [1])	Sibumasu [37]
	Indochina [46], [50], [51]
	Sukhothai including Lincang and Chanthaburi [42], [44], [48], [49], [52], [53], [54], [55], [56], [57]
	Inthanon Zone including Bentong Raub [44], [47], [49]
	Lhasa [58]
	Truong Son [59], [60]
Lead	[41], [59], [61], [62], [63].
^207^Pb/^204^Pb_(i)_ against ^206^Pb/^204^Pb_(i)_	
(Fig. 5d in [1])	
Grid of εNd_(t)_ including converted εHf_(t)_	[Published data from [9], [37], [38], [40], [44], [46], [48], [49], [51], [52], [55], [60], [64], [65], [66], [67]].
(Fig. 6 in [1])	
